# Application of digitization and visualization-based muscle strength measurement in ischemic stroke patients with motor dysfunction

**DOI:** 10.1038/s41598-023-44826-9

**Published:** 2023-10-16

**Authors:** Zhongyu Ren, Shangrong Ye, Qi Nie, Junlin Feng, Kaixiang Liu, Qinghua Li, Jian Wen

**Affiliations:** 1 Nervous System Clinical Medicine Research Center, Affiliated Hospital of Guilin Medical University, Guilin, 541001 China; 2 Department of Neurology, Affiliated Hospital of Guilin Medical University, Guilin, 541000 China; 3 Renmin Hospital of Wuhan University, Wuhan, 430000 China

**Keywords:** Stroke, Neurological disorders, Movement disorders

## Abstract

Ischemic stroke stands as a prevalent neurological ailment, where a paucity of methodologies exists for the assessment of functional outcomes post-stroke. Our objective was the development of a WeChat applet for the evaluation of muscle strength and the subsequent evaluation of its validity in ischemic stroke patients experiencing motor dysfunction. The assessment encompassed Lovett and NIHSS, followed by muscle strength values (MSV) and muscle strength ratios (MSR). These metrics were then scrutinized in relation to NIHSS and Lovett, followed by examination of their interrelationships. We enrolled a cohort of 157 patients, with an average age of 65 years, consisting of 96 males and 61 females. Lovett scores in the range of 2–4 and NIHSS scores spanning from 0 to 3 were found to correspond to specific values of MSV and MSR, respectively. Upon conducting correlation analysis, we noted that both MSV and MSR exhibited significant positive correlations with Lovett scores and NIHSS. Remarkably, the correlation of MSR with Lovett scores or NIHSS surpassed that of MSV. The WeChat applet offers a means of digitization and visualization of muscle strength. It correlates well with Lovett score and NIHSS, especially MSR. This bears potential significance in guiding the rehabilitation of stroke patients.

## Introduction

Stroke stands as the most prevalent neurological disorder affecting middle-aged and elderly populations, ranking as the foremost cause of disability among individuals aged 40 and older^1^. As our society ages, the incidence of stroke is on the rise, affecting approximately 16 million people worldwide^2,3^. Despite the largely preventable nature of strokes, as evidenced by decreasing incidence rates in various countries over the years, a substantial 70% to 80% of stroke survivors experience 'residual impairments and activity limitations'^4,5^. Among which motor dysfunction takes center stage^6^. More than two-thirds of post-stroke individuals grapple with challenges in 'upper limb function,' which can curtail one's ability to carry out various daily activities, hinder productivity and curtail social engagement. Consequently, this places a significant burden on patients, their families and society at large^7,8^.

Ischemic stroke accounts for approximately 60–80% of all stroke cases and represents a significant contributor to severe, long-term disability and mortality^9,10^, establishing its increasing relevance in the realm of future neurology. Randomized controlled clinical trials have unequivocally demonstrated the advantages of early endovascular intervention in reducing both mortality rates and post-stroke complications^11–16^. The implementation of suitable secondary prevention measures assumes paramount importance in enhancing the health outcomes of individuals recovering from a stroke. Moreover, upper limb hemiparesis emerges as the prevailing disability among stroke survivors^17^. Thus, timely assessment of muscle strength holds critical significance for early evaluation, treatment efficacy monitoring, and post-stroke rehabilitation^18^.

Among the parameters utilized to evaluate muscle condition, including muscle strength, muscle volume, and muscle density, muscle strength consistently stands as the most frequently employed metric. It serves as an effective means to glean insights into neural deficits, distinguishing genuine weakness from imbalances or diminished endurance. Its significance has been steadily gaining recognition^19–21^. Notably, the deltoid and trapezius muscle groups assume primary responsibility for arm lifting and abduction, rendering them pivotal in the assessment of upper extremity strength. Hence, we elected to focus our assessment on these muscle groups. Existing systems for evaluating upper limb mobility encompass the Lovett score, the Upper Limb Movement section of the National Institute of Health Stroke Scale (NIHSS), the verbal guidelines of the Fugl-Meyer Assessment (FMA), the Motor Function Status Scale, and the Upper Limb Movement Research Scale. Each evaluation system exhibits its unique merits and drawbacks, with the assessment process mandating patients to execute specific commands or actions. Consequently, assessment outcomes remain susceptible to an array of unpredictable variables, including the assessor's expertise, methodology, and the patient's condition. Portable muscle strength assessment devices have already made their debut, exemplified by the outcomes of Romero-Franco's 2019 research^22^. These devices offer cost-effectiveness and a judicious balance between efficiency and reliability, all while retaining portability. They afford the convenience of conducting muscle strength assessments at any juncture, thereby serving as a boon to professionals. In addition to portability, our device aligns itself more closely with the unique needs of patients and their respective conditions. It addresses specific limitations inherent to the Lovett score and NIHSS evaluations, further establishing a Bluetooth connection to the WeChat mini-program for seamless data storage. This feature streamlines the monitoring of changes in patients' conditions. Concurrently, through cloud-based data transmission, it facilitates data sharing between patients and healthcare providers, significantly curtailing the squandering of both space and time resources. Consequently, our research endeavors to introduce an improved, effective, evidence-based approach for appraising muscle strength, leveraging the WeChat applet for muscular strength assessment while concurrently assessing its construct validity. This approach builds upon existing technology to redress the inadequacies of current methodologies.

## Patients and methods

### Participants

An observational cross-sectional study was conducted between January 2020 and January 2021 to assess muscle strength in ischemic stroke patients experiencing motor dysfunction. Participants were recruited exclusively from the Department of Neurology at the Affiliated Hospital of Guilin Medical College in Guilin, China. Relevant patient data, encompassing age, gender, presence of comorbidities such as hypertension, diabetes, and coronary artery disease, were systematically extracted from the hospital's medical records. To mitigate potential biases, standardized measurements were consistently administered at a fixed daily time slot (evening, 18:00–20:00). Sample size determination for this study was guided by existing literature^23^, with concerted efforts undertaken to maximize patient enrollment. Inclusion criteria for the final study encompassed patients who met the following conditions: (1) a confirmed diagnosis of ischemic stroke via imaging modalities (brain CT or MRI) and accompanying neurological deficits characterized by unilateral limb movement impairments; (2) availability of comprehensive clinical data, the ability to fully participate in the study, a Lovett score ranging from 2 to 4, and an "Upper Limb Movement" section of the NIHSS score ranging from 0 to 3, without any impediments preventing arm raising or abduction, such as shoulder impingement; (3) informed consent provided by patients and their families, coupled with a clear understanding of the experimental process. Conversely, individuals with physical disabilities or other conditions adversely impacting the affected upper limb, severe cognitive impairment, and serious cardiac issues were excluded from the study. All clinical investigations were conducted in strict adherence to the principles outlined in the Declaration of Helsinki, and the study protocol received ethical approval from the Ethics Committee of Guilin Medical University (Protocol ID: YJSLL202132).

### Evaluation of Lovett muscle score

Two highly skilled neurosurgeons, who underwent specialized training and adhered to a meticulously standardized measurement protocol, conducted assessments of the patients' Lovett score, NIHSS, and muscle strength value. Patients were positioned supine and instructed to raise their arm at a 45-degree angle. Lovett scores were meticulously assigned in accordance with predefined scoring criteria, initially on the unaffected side and subsequently on the affected side. Only the value recorded on the affected side was integrated into the final analysis and reported. As elucidated in a prior study, the Lovett scale encompasses six distinct grades, each indicative of varying levels of muscle strength^24^. Within this framework, a score of 0 signifies the absence of observable voluntary muscle contraction, while a score of 5 signifies normal muscle strength, exemplified by a patient capable of overcoming resistance.

### Evaluation of "upper limb movement" section of NIHSS score

The NIHSS, often employed as an early secondary outcome measure in stroke trials, yields scores spanning from 0 to 42, with higher scores signifying more pronounced neurological deficits^25^. In this context, the 'Upper Limb Movement' section of the NIHSS score was administered to assess the muscle strength of each ischemic stroke patient. The specific methodology entailed placing the patient in a supine position and instructing them to elevate their arm to a 45-degree angle. Their capacity to sustain this position for a duration of 10 s was diligently observed, with scores subsequently allocated based on predetermined criteria. Assessments were initially conducted on the unaffected side and then replicated on the affected side. Only the score originating from the affected side was integrated into the final analysis and subsequently reported.

### WeChat Applet for muscle strength measurement

**Figure 1:**
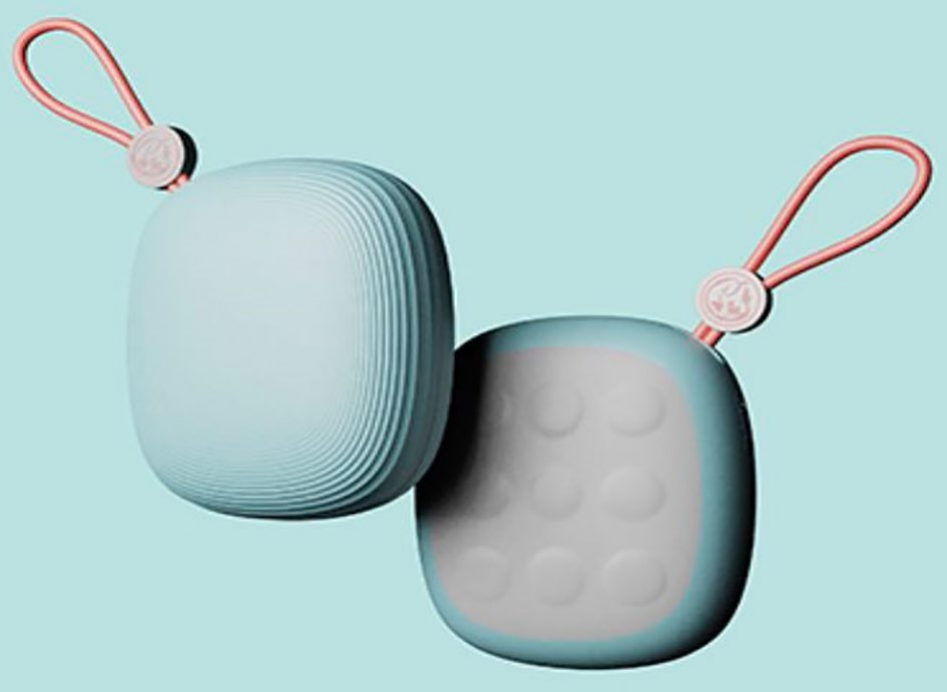
Schematic diagram of the instrument.

**Figure 2:**
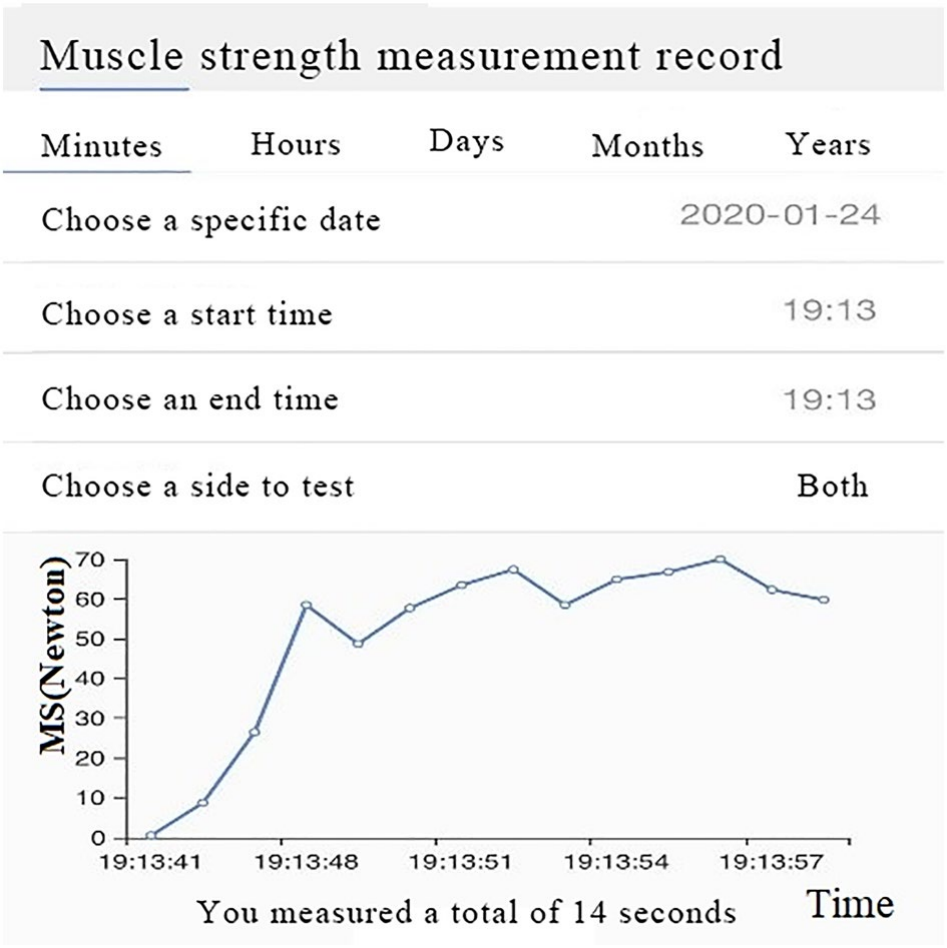
WeChat Applet Panel—14-second muscle strength measurement record.

Following the completion of Lovett score and NIHSS assessment, patients underwent a 5-min resting period. Subsequently, upper limb muscle strength was quantified utilizing the WeChat mini-program, yielding measurements for both MSV and MSR. To commence, the assessor provided a standardized demonstration. Subsequently, with the patient in a supine position, the outer elbow of the upper limb was positioned on the force measuring surface of the dynamometer, with the back resting on the immobilizer (weighting, 300 g, dimensions: 9.5 * 9.5 * 4.5 cm, Patent No. 202011218709.0, manufactured by Guangxi Jiugaohe Intelligent Technology Co., Ltd.) (Fig. 1). The patient was then instructed to exert maximal force in abducting the upper limb on the unaffected side. Once the muscle strength values stabilized on the display screen, MSVs recorded within a 10-s interval were documented, and the mean value was subsequently calculated as the final MSV (Fig. 2). Similarly, the muscle strength of the affected-side upper limb was assessed using identical procedures. Subsequently, the patient's 'affected to unaffected upper limb' MSR was computed using the formula ((MSR [%] = $\frac{{{\text{MSV}}\;{\text{of}}\;{\text{ the}}\;{\text{ affected}} - {\text{side}}\;{\text{ upper }}\;{\text{limp}}}}{{{\text{MSV }}\;{\text{of}}\;{\text{ the}}\;{\text{ unaffected}} - {\text{side }}\;{\text{upper }}\;{\text{limp}}}} \times 100{ }$).). Only the MSV or MSR originating from the affected side was integrated into the final analysis and subsequently reported.

### Ethical approval

This research received approval from the Ethics Committee of the Affiliated Hospital of Guilin Medical University, and we meticulously adhered to ethical standards throughout the study.

## Statistical analysis

Continuous data were presented as mean ± standard deviation. Group comparisons were conducted using one-way analysis of variance (ANOVA), followed by Bonferroni’s post hoc test to identify intergroup differences. Categorical data were expressed as percentages. The chi-squared test was employed to assess variations in gender, age, affected side, height, and weight distributions across different Lovett scores and NIHSS scale levels. This approach was utilized to mitigate potential bias stemming from these factors. Upon grouping in accordance with Lovett scores or NIHSS, suitable analytical methods were chosen for correlation analyses involving MSV and MSR, following a validation of data normality. All statistical analyses were executed using SPSS Statistical Software version 23.0 (SPSS, Inc., Chicago, IL, USA). All reported *P* values were two-tailed, with statistical significance defined as *P* < 0.05.

## Results

### Patient characteristics

**Figure 3:**
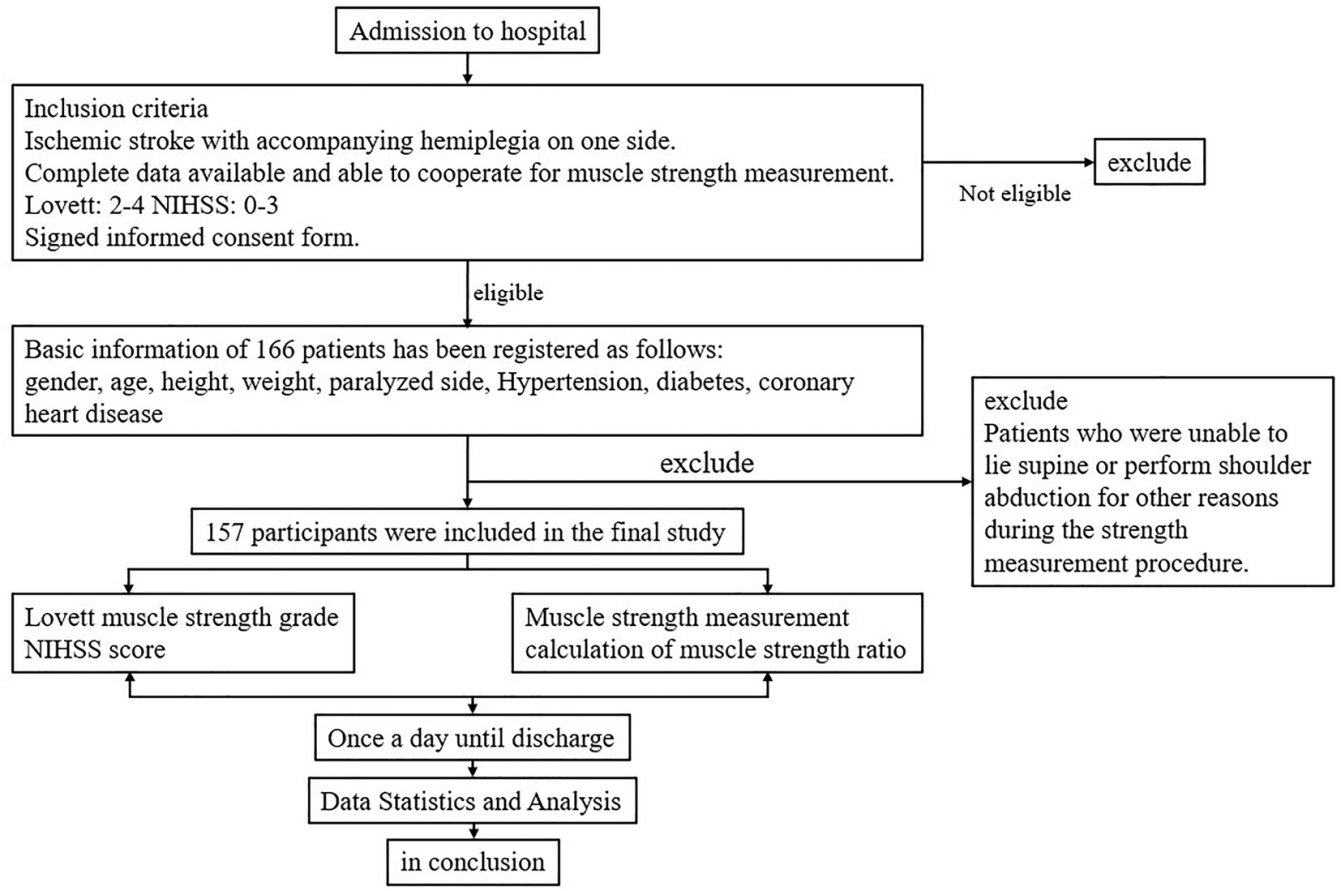
Study flow chart and participant enrollment results.

Based on the aforementioned criteria for inclusion and exclusion, a total of 157 ischemic stroke patients with motor impairment were enrolled in this study (Fig. 3). Their mean age stood at 65 years, while their average height and weight were recorded as 163 cm and 64 kg, respectively. Upon classification according to Lovett score or NIHSS, it is noteworthy that there existed no statistically significant disparities in the distribution of these factors among the respective subgroups (Table 1 and Table 2; both *P* > 0.05). This observation underscores the conclusion that the distribution of these factors within subgroups, encompassing gender, age, height, weight, and the affected side, exerts no influence on the comparative analysis of muscle strength across distinct subgroups.

**Table 1:** Demographic characteristics of patients grouped by Lovett muscle score [n (%)].

Characteristics	Lovett muscle score*			χ^2^	*P* value
	G2	G3	G4		
Gender					
Male	28 (66.7)	25 (52.1)	43 (64.2)	2.458	0.293
Female	14 (33.3)	23 (47.9)	24 (35.8)		
Age					
≤ 65	22 (52.4)	24 (50.0)	35 (52.2)	0.070	0.965
> 65	20 (47.6)	24 (50.0)	32 (47.8)		
Side					
Right	24 (57.1)	24 (50.0)	31 (46.3)	1.224	0.542
Left	18 (42.9)	24 (50.0)	36 (53.7)		
Height					
≤ 163	22 (52.4)	23 (47.9)	32 (47.8)	0.256	0.88
> 163	20 (47.6)	25 (52.1)	35 (52.2)		
Weight					
≤ 64	21 (50.0)	18 (37.5)	33 (49.3)	1.952	0.377
> 64	21 (50.0)	30 (62.5)	34 (50.7)		

G2–G4, Grade 2–4 of the Lovett muscle score.

*No patients with grades 0, 1, and 5 were involved.

**Table 2:** Demographic characteristics of patients grouped by NIHSS score [n (%)].

Characteristics	NIHSS score*				χ^2^	*P* value
	S0	S1	S2	S3		
Gender						
Male	43 (64.2)	11 (42.3)	14 (63.6)	28 (66.7)	4.739	0.192
Female	24 (35.8)	15 (57.7)	8 (36.4)	14 (33.3)		
Age						
≤ 65	35 (52.2)	14 (53.8)	10 (45.5)	22 (52.4)	0.406	0.939
> 65	32 (47.8)	12 (46.2)	12 (54.5)	20 (47.6)		
Side						
Right	31 (46.6)	11 (42.3)	13 (59.1)	24 (57.1)	2.567	0.463
Left	36 (53.7)	15 (57.7)	9 (40.9)	18 (42.9)		
Height						
≤ 163	32 (47.8)	14 (53.8)	9 (40.9)	22 (52.4)	1.054	0.788
> 163	35 (52.2)	12 (46.2)	13 (59.1)	20 (47.6)		
Weight						
≤ 64	33 (49.3)	10 (38.5)	8 (36.4)	21 (50.0)	1.973	0.578
> 64	34 (50.7)	16 (61.5)	14 (63.6)	21 (50.0)		

S0-3, 0–3 points of the NIHSS score.

*No patients with other NIHSS scores were involved.

### WeChat Applet for muscle strengthen measurement after grouping by Lovett muscle score

**Figure 4:**
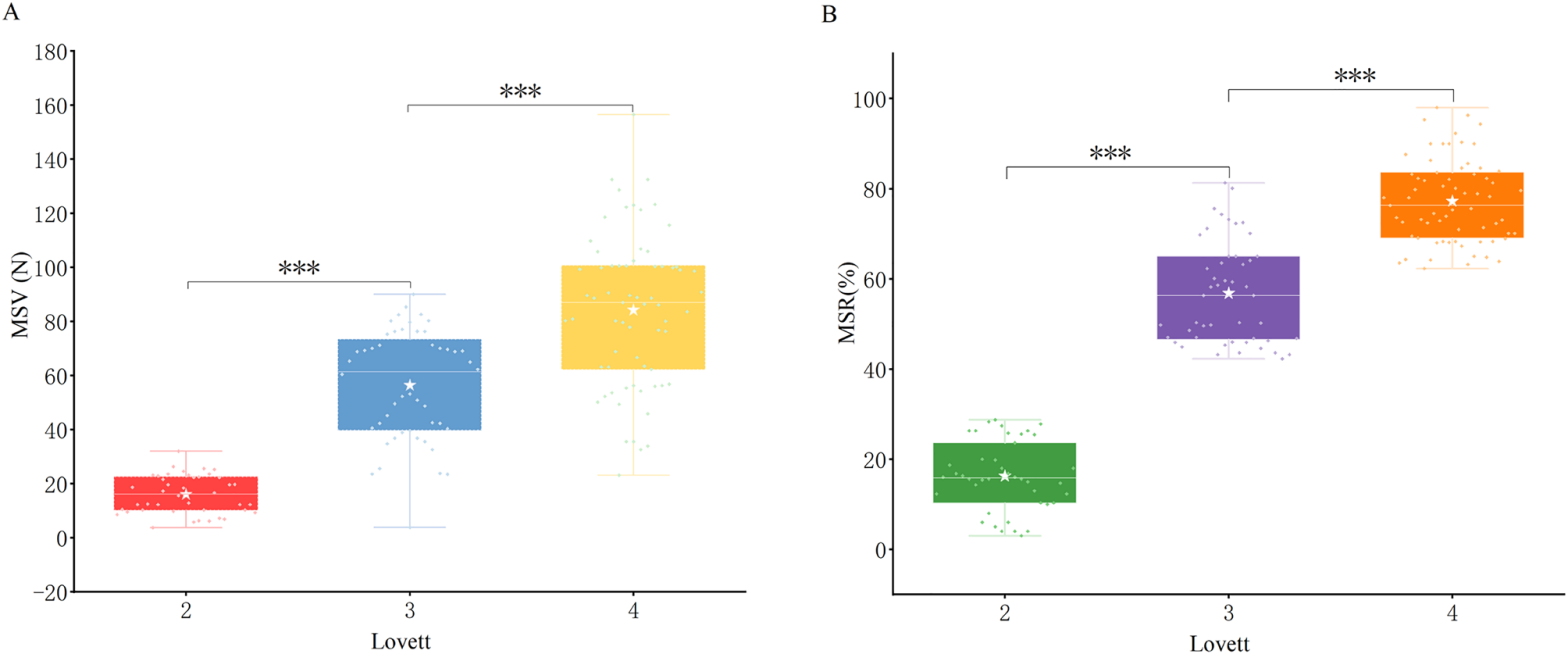
Box plot illustrating the distributions of MSV and MSR among patients grouped by Lovett muscle score. Notes: MSV, muscle strength value; MSR, muscle strength ratio. ^***^P < 0.001.

When grouped according to the Lovett score (Fig. 4A,B), the mean MSV of the affected-side upper limb exhibited the following values: 16.1 ± 7.1 Newton (N) with a 95% confidence interval (CI) ranging from 13.8 to 18.3 N for the 42 patients in grade 2; 61.3 ± 16.7 N (95% CI 56.1–66.5) and 58.9 ± 11.4% (95% CI 53.8–63.9%) for the 48 patients in grade 3; and 83.2 ± 26.1N (95% CI 75.0–91.3) and 77.2 ± 8.2% (95% CI 73.6–80.9%) for the 67 patients in grade 4. No patients were categorized under Lovett scores 0, 1, or 5. Furthermore, Welch’s test underscored statistically significant distinctions in the overall mean values of MSV and MSR across multiple groups (all *P* < 0.001). Subsequently, Bonferroni’s test revealed statistically significant differences in both MSV and MSR among patients with muscle strength grades 2, 3, and 4 (all *P* < 0.001) (Fig. 4). Upon testing the normality of the data, Spearman correlation coefficient analysis was executed. The outcomes unveiled significant correlations between both MSV and MSR with the Lovett muscle score, boasting correlation coefficients of 0.791 and 0.876, respectively (both *P* < 0.01).

### WeChat Applet for muscle strength measurement after grouping by NIHSS score

**Figure 5:**
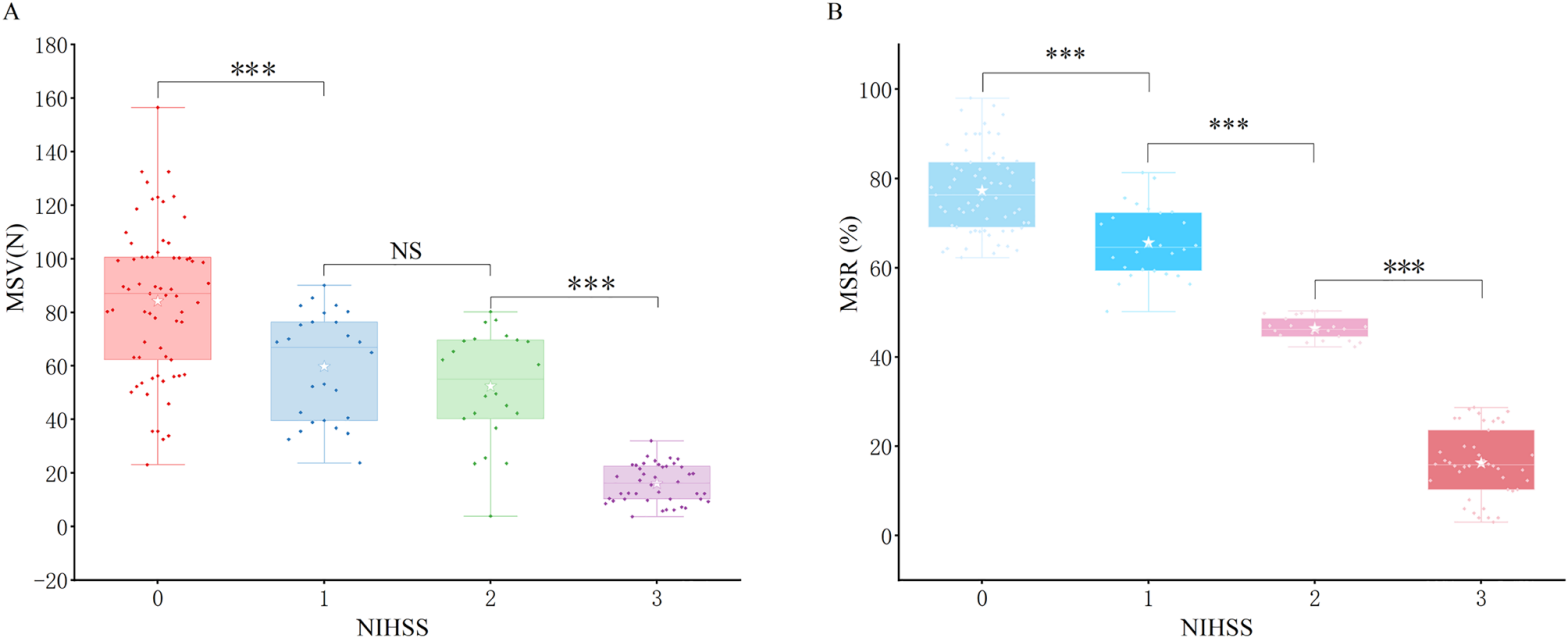
Box Plot Illustrating the Distributions of MSV and MSR grouped by NIHSS Score. Notes: MSV, muscle strength value; MSR, muscle strength ratio; NIHSS, National Institute of Health stroke scale. ***P < 0.001.

When categorized according to the 'Upper Limb Movement' section of the NIHSS (Fig. 5A,B), the mean MSV and MSR values were as follows: For 67 patients with an NIHSS score of 0: MSV was 88.0 ± 20.9 N (95% CI 78.7–97.2%) and MSR was 77.2 ± 8.2% (95% CI 73.6–80.9%). For 26 patients with an NIHSS score of 1: MSV was 64.9 ± 17.3 N (95% CI 57.2–72.6) and MSR was 66.8 ± 6.8% (95% CI 62.8–68.8%). For 22 patients with an NIHSS score of 2: MSV was 52.4 ± 21.0 N (95% CI 43.1–61.7) and MSR was 46.4 ± 2.5% (95% CI 45.3–47.5%). For 42 patients with an NIHSS score of 3: MSV was 14.3 ± 7.3 N (95% CI 11.0–17.5) and MSR was 16.5 ± 7.7% (95% CI 13.1–20.0%). As depicted in Fig. 4, statistically significant distinctions were evident in the overall mean MSV and MSR values when grouped by NIHSS score (all *P* < 0.001). Bonferroni post hoc testing revealed significant differences in MSV among the NIHSS 0, 2, and 3 subgroups (all *P* < 0.001), while no statistically significant differences were detected between the NIHSS 1 and 2 groups (*P* = 0.247). Conversely, significant statistical disparities were identified across all MSR subgroups based on NIHSS scores (all *P* < 0.001). Upon assessment of data normality, Spearman correlation coefficient analysis was conducted. The findings underscored noteworthy correlations between both MSV and MSR with the NIHSS score, yielding correlation coefficients of 0.791 and 0.899, respectively (both *P* < 0.01).

## Discussion

Early diagnosis and treatment of stroke are paramount for achieving favorable clinical outcomes^26^. The evaluation of muscle strength is an integral component of a patient's comprehensive assessment and plays a pivotal role in stroke diagnosis, treatment modality selection, and prognosis evaluation^27^. The utilization of the WeChat Applet, also known as a Mini Program, represents a straightforward and convenient tool accessible directly without the need for a separate app installation. In this study, we employed a WeChat Applet for digitization and visualization-based muscle strength measurement, aiming to ascertain alterations in muscle strength among post-stroke patients. This approach has the potential to reduce reliance on patient-reported scores and evaluation data, thereby enhancing objectivity and mitigating retrospective bias.The merits and innovations of this clinical trial are delineated as follows.

Given the multitude of methods available for assessing muscle function and physical performance, even seasoned clinicians often grapple with the challenge of selecting an appropriate tool validated for older populations, particularly those who have experienced a stroke^28^. Many of the highly specialized approaches necessitate professional training and tend to consume a significant amount of time during evaluation, as exemplified by the widely employed Lovett and NIHSS scoring systems^11,24^. These factors not only encumber healthcare providers but also impose constraints on patient cooperation. Furthermore, they do not facilitate the continuous, long-term assessment of muscle strength. Simultaneously, it is imperative to acknowledge that due to variations in the methodology and the patients' condition at the time of testing, the Lovett and NIHSS scores may not consistently align with the patients' actual muscle strength^29^. As evidenced by the research findings, the comparison of MSV in the NIHSS score 1 and 2 groups did not yield statistical significance (*P* = 0.247). This variability can be attributed to the inherent characteristics and behavioral state of the patients undergoing the muscle strength evaluation process. On occasion, during the measurement of muscle strength, patients may have been at rest or in a transitional state upon awakening from sleep. Consequently, the Lovett score, NIHSS score and MSV may not accurately reflect the patient's true condition. To address this, we introduced the assessment of MSR. Moreover, muscle strength measurement has exhibited substantial potential in assessing patient outcomes. Throughout the course of our research, we observed instances where some patients maintained a consistent Lovett Score level during their hospitalization, yet their MSV and MSR continued to rise. This observation suggests that within a narrow range of muscle strength fluctuations, the WeChat Applet employed for muscle strength measurement demonstrates greater sensitivity compared to Lovett score assessments. Consequently, Lovett score evaluations may not be ideal for gauging treatment efficacy in such patients.

While quantifying both MSV and MSR, we established a meaningful correspondence with Lovett and NIHSS scores, facilitating their validation and comparison. Upon analyzing the data, it is evident that both MSV and MSR exhibit commendable correlations with the Lovett and NIHSS score, with MSR demonstrating particularly strong associations. Figures 3 and 4 reveal that the SD of MSV is consistently relatively high, and there is a noticeable 'overlapping' phenomenon in the 'distribution interval of MSVs' between Lovett scores 3 and 4. This overlapping occurrence may compromise the accuracy of muscle strength assessment, underscoring the rationale behind our emphasis on MSR. In light of these considerations, we propose the utilization of MSR in conjunction with MSV for muscle strength assessment to enhance precision. In future investigations, we intend to explore the application of the 'maximum strength multiplied by duration' concept to further assess changes in patients' muscle strength.

Furthermore, owing to the absence of specialized training requirements, patients can autonomously conduct muscle strength measurements with the assistance of family members. Notably, each muscle strength measurement can be securely stored within the WeChat Applet's cloud database, forming an individualized repository. This database can be swiftly and systematically reviewed, offering valuable insights into the effectiveness of treatment. Patients also have the capability to share their data with physicians through cloud services, facilitating real-time monitoring of their condition. This, in turn, aids physicians in promptly devising personalized treatment strategies. Standardizing muscle strength assessment for healthcare professionals can be challenging, given variations in experience, skill levels, and understanding. However, the WeChat Applet for muscle strength mitigates this issue. Furthermore, using a single account, muscle strength data can be shared among healthcare providers with the patient's consent, significantly enhancing operational efficiency. Presently, online medical consultations have permeated various facets of daily life. The WeChat Applet for muscle strength empowers patients to transmit their muscle strength information to healthcare professionals without leaving their homes, conserving substantial time and space resources. Similarly, it assumes a pivotal role in facilitating inter-hospital consultations.

Nevertheless, our experiment is not without its limitations. Firstly, it is a single-center study of short duration, and the results may be influenced by individual factors. We did not assess the intra- and inter-inspector reliability of MSV and MSR, which is a limitation. Secondly, due to constraints in muscle strength and measurement range, the instrument is presently incapable of evaluating muscle strength in patients with lower Lovett muscle scores. Lastly, our exclusion criteria precluded the inclusion of patients with Lovett muscle scores of 0 and 1. Consequently, the development of new blood biomarkers is imperative to elucidate clinical intervention strategies pertinent to treatment efficacy in patients with lower Lovett muscle scores. As such, long-term multicenter experimental studies are essential for further validation.

## Conclusion

The WeChat Applet for muscle strength measurement exhibits superior quantitative sensitivity, enabling the digitization and visualization of muscle strength in ischemic stroke patients. To some extent, it correlates reasonably well with both the prevailing Lovett muscle score and the 'Upper Limb Movement' section of the NIHSS score. Consequently, it carries substantial implications for clinicians in guiding clinical treatment decisions and assessing the prognosis of ischemic stroke patients experiencing motor dysfunction. In conclusion, it merits further exploration through clinical research, broader application and promotion as a valuable tool for muscle strength assessment and treatment efficacy evaluation.

### Supplementary Information

Supplementary Information.

## Data Availability

The datasets used and/or analyzed during the current study are available from the corresponding author upon reasonable request.
